# A model-based analysis of impulsivity using a slot-machine gambling paradigm

**DOI:** 10.3389/fnhum.2014.00428

**Published:** 2014-07-03

**Authors:** Saee Paliwal, Frederike H. Petzschner, Anna Katharina Schmitz, Marc Tittgemeyer, Klaas E. Stephan

**Affiliations:** ^1^Translational Neuromodeling Unit (TNU), Institute for Biomedical Engineering, University of Zurich and Swiss Federal Institute of Technology (ETH Zurich)Zurich, Switzerland; ^2^Max Plank Institute for Neurological ResearchCologne, Germany; ^3^Laboratory for Social and Neural Systems Research (SNS), University of ZurichZurich, Switzerland; ^4^Wellcome Trust Centre for Neuroimaging, University College LondonLondon, UK

**Keywords:** Hierarchical Gaussian Filter, Hierarchical Bayesian Model, Barratt Impulsiveness Scale, impulsivity, pathological gambling

## Abstract

Impulsivity plays a key role in decision-making under uncertainty. It is a significant contributor to problem and pathological gambling (PG). Standard assessments of impulsivity by questionnaires, however, have various limitations, partly because impulsivity is a broad, multi-faceted concept. What remains unclear is which of these facets contribute to shaping gambling behavior. In the present study, we investigated impulsivity as expressed in a gambling setting by applying computational modeling to data from 47 healthy male volunteers who played a realistic, virtual slot-machine gambling task. Behaviorally, we found that impulsivity, as measured independently by the 11th revision of the Barratt Impulsiveness Scale (BIS-11), correlated significantly with an aggregate read-out of the following gambling responses: bet increases (BIs), machines switches (MS), casino switches (CS), and double-ups (DUs). Using model comparison, we compared a set of hierarchical Bayesian belief-updating models, i.e., the Hierarchical Gaussian Filter (HGF) and Rescorla–Wagner reinforcement learning (RL) models, with regard to how well they explained different aspects of the behavioral data. We then examined the construct validity of our winning models with multiple regression, relating subject-specific model parameter estimates to the individual BIS-11 total scores. In the most predictive model (a three-level HGF), the two free parameters encoded uncertainty-dependent mechanisms of belief updates and significantly explained BIS-11 variance across subjects. Furthermore, in this model, decision noise was a function of trial-wise uncertainty about winning probability. Collectively, our results provide a proof of concept that hierarchical Bayesian models can characterize the decision-making mechanisms linked to the impulsive traits of an individual. These novel indices of gambling mechanisms unmasked during actual play may be useful for online prevention measures for at-risk players and future assessments of PG.

## Introduction

Uncertainty is a fundamental aspect of human decision-making (Bland and Schaefer, 2012). One general framework for assessing decision-making under uncertainty is to view humans as Bayesian learners. From this perspective, humans employ a generative model of sensory inputs to update beliefs about the state of the world and choose actions in order to minimize prediction errors (Knill and Pouget, 2004; Daunizeau et al., 2010; Friston et al., 2010). When this predictive machinery breaks (due to disease or drugs), maladaptive behavior can arise. This aberrant behavior can be formally examined and understood mechanistically using different computational models (e.g., McGuire and Kable, 2013). One interesting and clinically relevant case of potentially harmful aberrant behavior that arises is impulsivity, i.e., actions without deliberation or forethought, particularly in the face of uncertainty (Dickman, 1993; Sharma et al., 2014).

Impulsive responses under uncertainty play a crucial role in disordered gambling, where players continue to bet money even in the face of large losses and potentially catastrophic long-term consequences. It has been found that standard measures of impulsivity and gambling severity scores are significantly correlated (Alessi and Petry, 2003; Krueger et al., 2005). Pathological gambling (PG) was therefore originally categorized as an “Impulse Control Disorder Not Elsewhere Classified” in the Diagnostic and Statistical Manual (DSM) Fourth Edition. It has recently been relabeled “gambling disorder” and reclassified as an addictive disorder in the 5th edition of the DSM, due to the large number of characteristics it shares with other addictions. This, however, does not question the relationship between impulsivity and disordered gambling, since impulsivity is a central theme in addiction as well (Holden, 2010; APA, 2013).

Impulsivity has been shown to have predictive power in assessing a subject's susceptibility to addiction (deWit, 2009; Leeman et al., 2014). In the specific context of gambling, correlations between gambling severity and more traditional questionnaire-based measures of impulsivity, such as the Eysenck's Impulsivity Inventory, the Barratt Impulsiveness Scale (11th version; BIS-11), the Urgency, Premeditation, Perseverance and Sensation-Seeking (UPPS) scale, and the Dickman Impulsiveness scale, have been reported (Monterosso and Ainslie, 1999; Rodriguez-Jimenez et al., 2006; Whiteside and Lynam, 2009). More specifically, changes in gambling severity were related to changes in self-reported impulsivity scores (Blanco et al., 2009). Given this evidence, impulsivity has been proposed as a potential predisposing factor for PG (Vitaro et al., 1997; Brewer and Potenza, 2008; Guerrieri et al., 2008; Stein, 2008).

However, impulsivity is a broad concept with many facets, including spontaneous acts without planning or deliberation (“acting without thinking”), excessive risk-taking, and a lack of orientation to future outcomes (Patton et al., 1995; Robbins et al., 2012). It is therefore conceivable that PG behavior involves only a subset of these elements (Nower and Blaszczynski, 2006). For example, response impulsivity (also referred to as “stopping impulsivity”; Robbins et al., 2012), measured by deficits in inhibitory control, showed mixed results when tested for in pathological and problem gamblers. Lawrence et al. (2009) found deficits in a Stop Signal Reaction Time Task (SSRT) with relation to alcohol dependence but not in relation to gambling behaviors. Similarly, Rodriguez-Jimenez et al. (2006) found decreased SSRT accuracy only in gamblers also diagnosed with Attention Deficit Hyperactivity Disorder (ADHD). Other studies, however, report that pathological gamblers commit more commission errors in a Go/NoGo task, indicative of impulse control problems (Fuentes et al., 2006).

By contrast, measures of choice impulsivity (or “waiting impulsivity”; Robbins et al., 2012) show a more consistent relation to gambling behavior. For example, higher discount rates in delay discounting tasks have been associated with problem and PG in a number of studies (Petry, 2001; Alessi and Petry, 2003; Peters and Büchel, 2011; Miedl et al., 2012). These deficits correlate mainly with cognitive distortions, suggesting that differences in the underlying belief structure of a gambler might contribute to the types of impulsivity we see in disordered gambling (Michalczuk et al., 2011). These findings are in line with reported decision-making deficits of gamblers across a variety of tasks (Goudriaan et al., 2005). This does, however, not explain how different cognitive mechanisms related to impulsivity translate into different gambling behaviors, from the recreational to the pathological.

Classical analyses of impulsivity, in the context of gambling, rest primarily on questionnaires (Eysenck and Eysenck, 1977; Barratt, 1985; Monterosso and Ainslie, 1999; Whiteside and Lynam, 2009). For many complex traits or psychological constructs (including impulsivity), self-report measures from questionnaires represent a gold standard. However, as they provide a descriptive summary of processes that may be controlled by factors not accessible through conscious introspection, they can be subject to various confounds (Wilson and Dunn, 2004). A promising alternative approach is to directly engage the subject in a paradigm that unmasks pathological behavior and apply a model that infers on the latent mechanisms underlying this behavior. This notion is rapidly gaining attention, particularly in the application to psychiatry (cf. “computational psychiatry”; Moutoussis et al., 2011; Montague et al., 2012; Stephan and Mathys, 2014), and represents the approach pursued in this paper. To gain acceptance in the field, however, any model-based approach of this sort will have to show construct validity with respect to an established standard, i.e., a commonly used questionnaire (for a similar rationale, see Huys et al., 2012). In our case, the BIS-11 represents one such widely accepted standard way of assessing impulsivity, and we thus used this questionnaire as a reference point for demonstrating the plausibility of our model-based characterization of impulsivity.

Formal modeling of the time series of responses during gambling (whether pathological or not) has received surprisingly little attention (one significant exception being Ligneul et al., 2012). However, there have been several publications in the recent past urging the community toward cognitive models of problem gambling (i.e., Gobet and Schiller, 2011). Some analyses have been motivated conceptually by reference to reinforcement learning (RL) (Shao et al., 2013), but we are not aware of studies that have directly applied a reinforcement-learning model to slot machine gambling data. This may be because classical RL does not directly relate to probabilistic inference on hidden states of the world *per se* (e.g., states of slot machines) but assumes states and actions to be given and accessible (Gershman and Niv, 2010). This lack of an intrinsic concept of uncertainty (with respect to states of the world) is not ideal for studying gambling behavior (Averbeck et al., 2013; McGuire and Kable, 2013). This suggests the application of Bayesian approaches, for which uncertainty is a core quantity. Wetzels et al. (2010), for instance, use an Expectancy Valence (EV) model to parameterize how subjects perceive wins and losses when engaging in the Iowa Gambling Task (IGT), and argue for the use of Bayesian cognitive models to explain gambling behaviors. Similarly, a recent call for increasing the role of mathematics in the psychological intervention in problem gambling highlights the need for further modeling approaches (Barboianu, 2013).

To yield mechanistic insights into gambling, we need to infer, from measured behavior, the principles that govern an individuals' belief-updating processes. This can be achieved using a Bayesian model of cognitive processes–one that illustrates how sequences of latent states and their respective uncertainties are transformed into observable responses. Bayesian models thus allow for “triple inference,” with respect to perception (inference on states of the world), learning (estimating the parameters that govern perceptual updates) and decision-making (the transformation of beliefs into actions). These quantitative estimates provide a more complete and mechanistically interpretable explanation of behavior in an individual, reflecting perceptual and decision-related nuances that simple summary statistics, such as average accuracy or reaction time, may have hidden from the experimenter (Mathys et al., 2011).

In the present work, we treat the player as an (approximate) Bayes-optimal learner who invokes a hierarchical generative model of trial outcomes in order to infer on the probabilistic structure of the game, allowing for optimal decisions under uncertainty (cf. Daunizeau et al., 2010). Having seen a trial outcome, the player updates his beliefs about trial-wise probabilities of winning and how these change in time (i.e., whether the slot machine is stable or volatile). Critically, these updates exhibit individual approximate Bayes-optimality (Mathys et al., 2011), governed by subject-specific parameters that couple the hierarchical levels of inference in the model. On any given trial, the ensuing beliefs then provide a basis for a response model that prescribes a probabilistic mapping from beliefs to responses.

A likely reason as to why there have been few attempts at formal modeling of slot machine gambling may be that it is not immediately obvious which of the many data features a naturalistic slot machine paradigm affords should be used to formulate a model for optimally predicting impulsivity (both in terms of sensory inputs and motor responses). Notably, this cannot be decided by standard statistical model comparison techniques since this requires the data to be constant across models. Here, we address this problem by examining construct validity. That is, for different combinations of sensory and motor data features, we assess the predictive power of the resulting model parameter estimates in relation to an external and independent variable.

To summarize, in this proof of concept study we evaluated the potential utility of a model-based approach to characterizing gambling behavior, combining a naturalistic gambling paradigm with generative (Bayesian) modeling to quantify gambling-relevant aspects of impulsivity. For this, we sought to establish construct validity in relation to standard questionnaire measures of impulsivity. Specifically, we first tested 48 male participants using a naturalistic slot-machine gambling paradigm task where a variety of different gambling behaviors could be expressed. We assessed the behavioral correlates in gambling behavior with respect to the individuals' impulsivity, as assessed by the BIS-11 (Patton et al., 1995) and independently modeled participants' belief-updating mechanisms by a hierarchical Bayesian framework (Hierarchical Gaussian Filter, HGF). Finally, we examined whether the model parameter estimates would predict the individuals' impulsive traits (BIS-11 scores).

## Materials and methods

### Experimental procedure

#### Participants

Participants included 48 healthy male subjects (Table 1). All volunteers gave written informed consent. The study was approved by the ethics committee of the Faculty of Medicine at the University of Cologne, Germany (study number 10-226). One subject left the task early, and was excluded from the analyses.

**Table 1:**
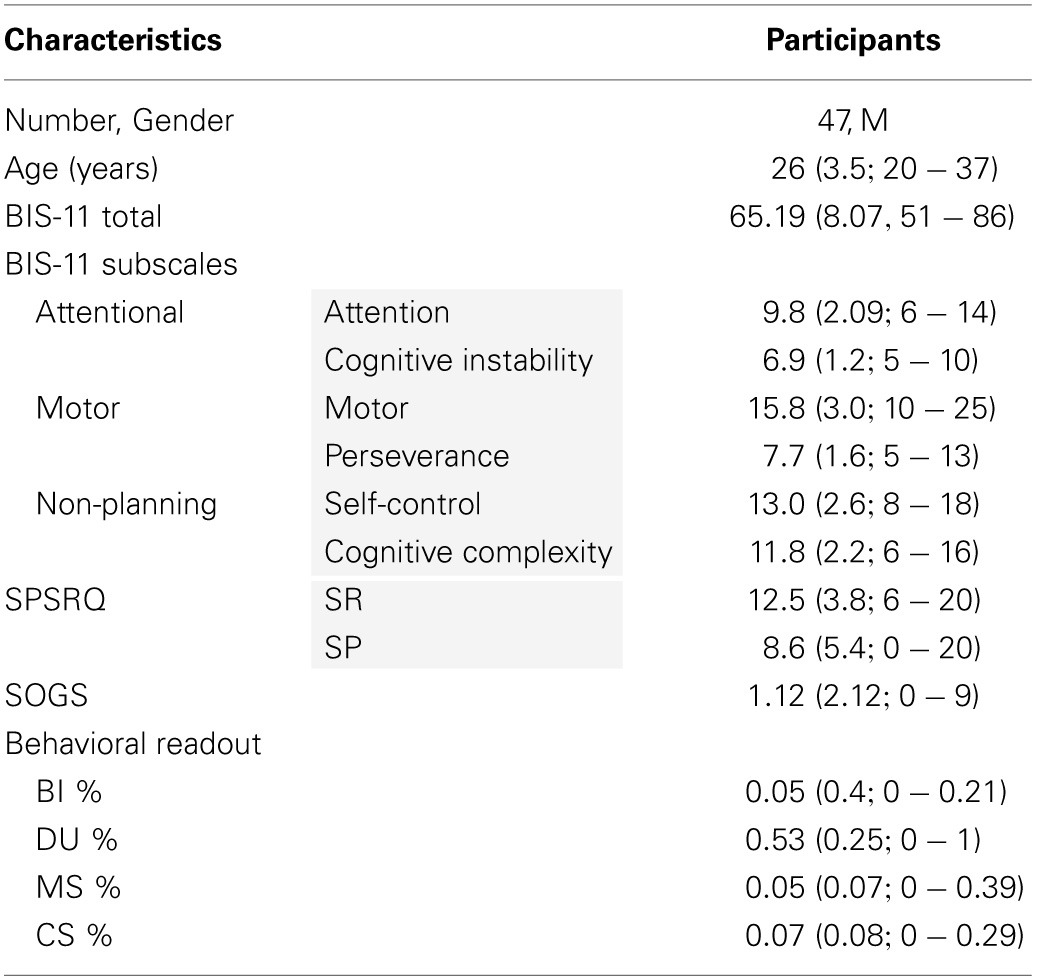
**Descriptive statistics**.

Group means are reported with standard deviation and range in parentheses. BIS-11, Barratt Impulsiveness Scale (maximum score: 120); SPSRQ, Sensitivity to punishment and reward questionnaire; SR, Sensitivity to Reward; SP, Sensitivity to Punishment (maximum scores: 24); BI%, Bet increase percentage; DU%, Double-up percentage (in cases a double-up was offered); MS%, Machine switch percentage, CO%, Casino switch percentage.

#### The Barratt Impulsiveness Scale

The Barratt Impulsiveness Scale (version 11; BIS-11) was used as an independent measure of impulsivity. It has a 50-year track record in psychiatric diagnosis and was validated as a measure of impulsivity in a series of studies (Moeller et al., 2001; Stanford et al., 2009). Here, we used the German version of BIS-11 (Patton et al., 1995). The BIS-11 is a 30-item self-report questionnaire divided into 6 first-order and 3 second-order sub-scales (First-order sub-scales: Attention, Motor, Self-control, Perseverance, Cognitive Instability, Cognitive Complexity; Second-order subscales: Attentional, Motor, Non-planning). We used the total score as the external impulsivity measure in our analyses of construct validity of computational models.

#### The Sensitivity to Punishment and Sensitivity to Reward Questionnaire

The Sensitivity to Punishment and Sensitivity to Reward Questionniare (SPSRQ) assesses the Behavioral Inhibition System and the Behavioral Activation System in the two subscales Sensitivity to Punishment (SP) and Sensitivity to Reward (SR), respectively. The SR has been found to relate positively to the Eysenck's Impulsivity Inventory and also has a significant positive correlation with the Sensation-Seeking Scale (SSS) (Torrubia et al., 2001). Here, we use this as a complementary scale, in addition to BIS-11, to examine gambling; in contrast to other impulsivity questionnaires, such as the UPPS Impulsive Behavior Scale (Whiteside and Lynam, 2009), the BIS-11 lacks an isolated sensation-seeking subscale, which we account for by using the SPSRQ. In the context of gambling behavior the more relevant measure will be the Sensivity to Reward subscale.

#### The South Oaks Gambling Screen

The South Oaks Gambling Screen (SOGS) is a self-administered 20-item questionnaire to screen for clincial populations with problem and PG based on criteria stated by the third edition of the Diagnostic Statistical Manual (DSM III). We assessed the SOGS to account for potential confounds of PG behavior in our analysis of impulsive gambling. The clincal cut-off of the SOGS proposed by Lesieur and Blume (1987) is 5, while the cutoff poposed by Tolchard and Battersby (1996) is 10. The mean score of our healthy subjects was 1.12. 3 out of 47 subjects in our study exceeded a SOGS score of 5, none of the subjects exceeded a SOGS score of 10. As the SOGS has been reported to be overly sensitive for assessments of the general population with a false positive rate of 50% (Stinchfield, 2002), we decided to include all subjects into the main analysis. We do not find a significant correlation between the BIS-11 and the SOGS (*r* = 0.18, *p* = 0.2).

#### Slot-machine paradigm

We designed a naturalistic behavioral paradigm to approximate the experience of true casino gambling by simulating a simple Electronic Gambling Machine (EGM). In addition to flexibility of design and ease of play, the literature suggests that EGMs have a higher addiction potential than other gambling alternatives, and increased access to these machines may lead to an increase in gambling problems across the world, independent of cultural context (Dowling et al., 2005; Lund, 2009). For these reasons, a slot-machine experimental paradigm proved particularly appealing in eliciting impulsive behavior from our subjects.

**Figure 1:**
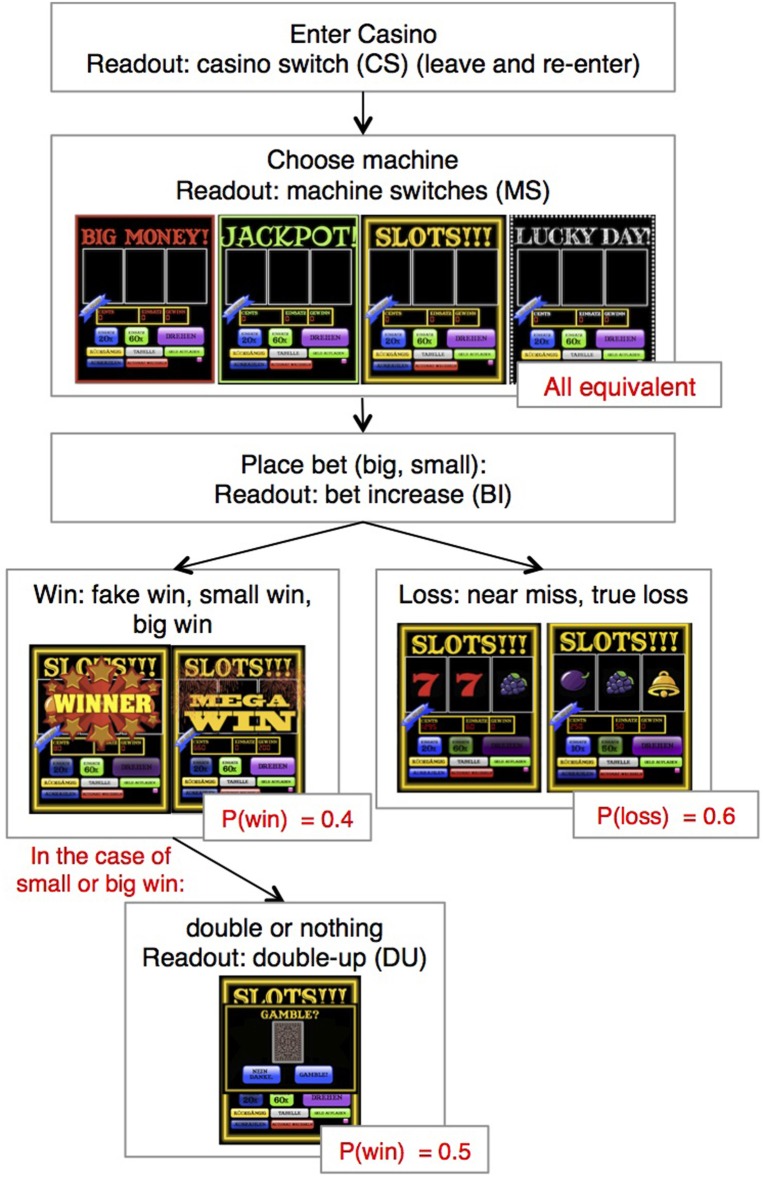
**Structure of slot machine game.** After introductory instructions and 5 training trials, the player “enters the casino.” He or she is then made to chose a machine, place a bet and pull the lever. As in a standard slot machine, the player watches the wheels spin and is then shown the trial outcome. On 50% of the win trials, the player is allowed to engage in a double-up option. He or she is given 3 seconds to decide whether or not to gamble. On any given trial, the player can also choose to switch the machine or switch between casino visits (not shown). The trial outcomes can be clarified as follows: “true wins” (small and big) were wins, in which the monetary amount won was larger than the original bet. “Fake wins,” were trials in which the monetary amount received was smaller than the original bet placed. “Near-misses” are trials in which the outcome of the trial was a loss, but only the last wheel was different (e.g., AAB), and true losses are trials in which the amount bet was greater than the amount won.

The features of the game, the design of slot-machines itself, and the probability trace and pay-out percentage were inspired by real slot machines in Swiss casinos, and allowed players significant freedom to express different types of gambling behavior (Figure 1). To increase engagement in the task, participants gambled with real money (20 Euros) that they symbolically received—in addition to their show-up fee—before the start of the slot-machine game. The actual payout (the sum of wins, losses, and the show-up fee) took place after the game was completed. With a view to future studies with PG patients, we designed the virtual slot machine to resemble a realistic one, in the hope that this will facilitate the emergence of underlying risk tendencies and allow us to measure a broad spectrum of potentially relevant behaviors. We used industry-typical color and sound effects to increase the subject's engagement in the task, making the experiment as entertaining and realistic as possible while keeping the response options sufficiently simple such that subjects without previous gambling experience could immediately understand the options of the game. From a modeling perspective, a casino-like game allows the experimenter to observe a rich set of behaviors that go beyond trial-wise bets. For example, the behavior and self-reports of many gamblers indicate that they are actively trying to estimate whether a given machine is “hot” (i.e., whether it is likely to produce wins) and switch to a new machine if it gets “cold” (Parke and Griffiths, 2006). To review such behavior, our paradigm offered four different slot machines, and the subject was free to switch casinos and switch machines at any point in time.

Generally, at any point during the game, the player was able to:

- switch between four different slot machines,
- switch casinos and re-enter the casino on a new virtual day,
- add money from wallet to machine,
- place small or large bets on each trial,
- check the scores of different fruit combinations,
- accept the double-up (DU) option after a subset of wins.

#### Behavioral readout

Participants played 200 trials of the slot machine gambling task, and could decide between 4 machines (see Figure 1) that differed in their visual appearance but not in choice options. Players had the option to switch between these machines at any point during the game (machine switch, MS). Similarly, they could also leave the virtual casino which would result in a cash-out of the money that was currently on the machine. After a casino switch (CS), participants had to reenter the casino on a “new day” in the virtual world (while the underlying probability structure of the trials, which was unknown to the subjects, continued) until they played through a minimum number of 200 trials.

At the beginning of the game, participants could decide how much of the money on their account they wanted to load into the slot machine and which of the four machines they wanted to play on. In each trial, they had to place one of two bets (low bet = 20 Cents, high bet = 60 Cents) before starting the spinning of all three wheels of the machine, one after another. After a delay of 1 s, the wheels stopped spinning and displayed a combination out of 9 possible stimuli, all of which were pre-programmed in the game. The magnitude of the win was determined by the combination depicted; possible wins ranged from 5 Cents to 60 Euro (the Jackpot win for a large bet). Participants could look up the win table at any point in time by pressing an extra button on the machine. After 50% of the wins, the player was offered a secondary gamble option, with a 50–50 chance of doubling or losing the win amount (Figure 1). The decision to accept the “DU” option had to be made within 3 s of the screen appearing, enforcing rapid decision-making.

#### Perceptual input

To differentiate between different types of learning, we used four different trial outcomes in the game: true wins, fake wins, near-misses, and true losses (compare Figure 1). “True wins” were wins, in which the monetary amount won was larger than the original bet, whereas in “fake wins,” the monetary amount received was smaller than the original bet placed. Fake wins have been found to reinforce the sense of winning in slot machine games, and were included here to identify whether these events play a role in characterizing impulsivity (Jensen et al., 2013). “Near-misses” refer to cases, where the bet was lost, but only the last wheel was different (e.g., AAB). This type of trial outcome has been shown to enhance gambling motivation, to lead to physiological arousal and to activate reward-related brain areas (Clark et al., 2009, 2012), all of which are related to subjective skill-oriented cognitive gambling traits, such as illusion of control (Billieux et al., 2012). Finally, “true losses” were cases in which all symbols depicted were different. Upon winning, the player experienced one of two different win banners (Figure 1), depending on the size of the win, where the distinction of “mega win” was reserved for the top three largest win amounts. In the case of fake wins, the same win-banner was shown to the player as for true “non-mega” wins.

#### Underlying game structure

To ensure comparable inference trajectories, each participant played the same sequence of 200 trials (with pre-determined win/loss outcomes), but was given the option to continue gambling past the end of this period if he desired. For comparison across subjects and modeling purposes, we analyse performance over the 200 trials only. Because the sequence of probabilities and reward levels were fixed across subjects, variability in performance could only result from the subject's own betting behavior and choices to engage in the DU option. Through systematic simulations, we chose a trace of probabilities and reward levels such that the mode of the return-to-player (RTP) for all types of potential bet combinations was around 90%, which is higher than the minimum required return to player of 70%, as stipulated by norms for actual casino RTPs (Gaming Laboratories International, 2011). The trace accounts for numerous relevant variables that may determine gambling behavior:

- 40% of the trials were wins,
- 50% of the wins were fake wins,
- 50% of the winning trials were followed by a DU option,
- 18% of trials were near misses,
- pre-determined winning and losing streaks.

**Figure 2:**
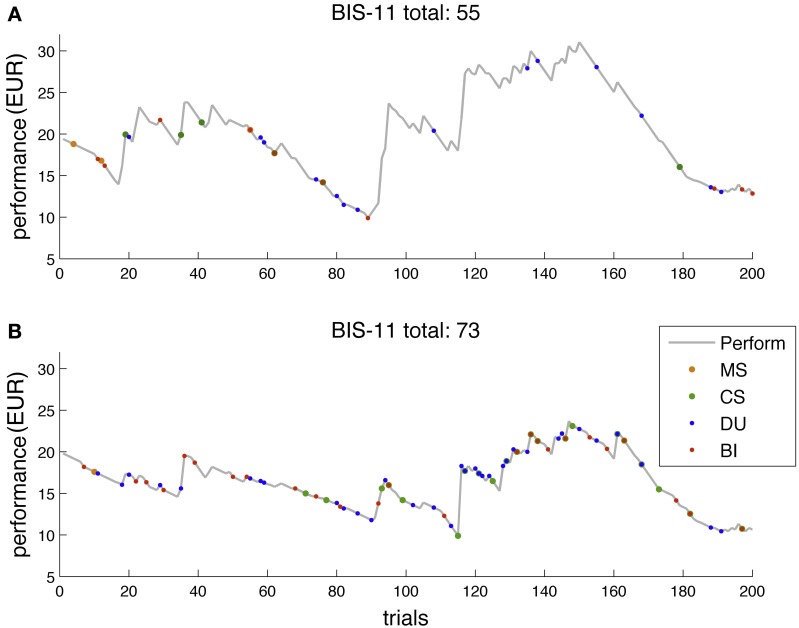
**Two exemplary performance traces for subjects with different BIS-11 scores. (A)** Subject with BIS-11 score below average (55). **(B)** Subjects with BIS-11 score above average (73). Gray trace, performance over the course of the game in EUR. Colored dots are overlayed to the performance trace and reflect events of interest in a particular trial. MS (orange), machine switch; CS (green), casino switch; DU (blue), double-up; BI (red), bet increase. Notably, the more impulsive a subject (i.e., the higher a subject's BIS score), the more the subject exhibited behavioral activation throughout the game, and in particular, in the more volatile phases of the paradigm.

Figure 2 depicts two exemplary performance traces for two subjects with BIS-11 score below (55) and above average (73). Behavioral readouts are overlaid in different colors. Notably, the more impulsive subject showed more behavioral activation and risk-seeking behavior throughout the game, in particular during the more volatile phase.

### Data analysis

In addition to computational modeling described below, we used classical multiple linear regression for analyzing the behavioral data and for evaluating the construct validity of computational models (i.e., testing relations between model parameter estimates and BIS-11 scores). These analyses were performed using the regstats function in the MATLAB Statistics Toolbox. To determine how much the variance of an estimated regression coefficient increased due to collinearity, we estimated the variance inflation factor (VIF) for each regressor. In all analyses, a *p*-value of < 0.05 was considered significant; multiple tests were corrected for by Bonferroni correction. For model comparison we used the Bayesian Information Criterion (BIC).

### Computational modeling

#### Generic considerations

This paper is concerned with a proof of concept demonstration that generative modeling of gambling behavior can yield mechanistic descriptions of impulsivity in terms of individual beliefs and belief-to-response mappings. A generative model is a model which provides a joint probability distribution over all random variables involved (e.g., observations and parameters). It specifies a forward mapping from hidden parameters and states to measurable observations. Here, we will consider generative models which are formulated under the “observing the observer” framework (Daunizeau et al., 2010). Such models allow the experimenter to infer upon the hidden states and parameters of an agent or subject engaged in a task.

Critically, this class of models generate two things: sensory inputs and motor responses. Therefore, when specifying a generative model of gambling, one must consider what aspects of the sensory input administered and the behavioral responses observed are to be predicted by the model. First, the player's internal belief updating (mediated by the “perceptual model”) could be informed by different aspects of trial outcomes to which he has sensory access (“perceptual variables”). For example, does he treat near-misses similar to wins of any sort, and does he distinguish between true wins and fake wins? Secondly, how is a given belief transformed into a behavioral response or choice? A particular belief-to-response mapping constitutes what we refer to as a “response model.” Importantly, in a naturalistic paradigm, many different aspects of behavior can be observed (e.g., bets, DUs, MS, etc.), and, similar to the perceptual variables above, a choice has to be made regarding what the most relevant data features are that should be predicted by the generative model. In other words, generative models could be constructed for different combinations of perceptual and response variables.

In principle, finding the optimal model can be accomplished by means of Bayesian model selection (BMS), which evaluates the relative plausibility of competing models in terms of the log evidence (MacKay, 2003) and represents a principled trade-off between model fit and model complexity. However, a condition for BMS is that the competing models predict identical data. This means that BMS can only proceed if both perceptual and response variables are identical.

To deal with this issue, we implement a two-stage model selection in this paper. As described in the next section, we consider five different “core models;” each of these represents a particular combination of a perceptual and a response model. We then consider three different perceptual variables and four response variables; this results in 12 sensory-motor datasets. For any of these datasets, we can invert all five core models and select an optimal model using BMS. In a second step, we can evaluate the relative goodness of these 12 selected models by assessing their construct validity against an external measure of impulsivity. To this end, we use an independent questionnaire-based measure of impulsivity (the BIS-11) and perform multiple regression analyses of individual parameter estimates on individual questionnaire scores, as described below.

Following this general overview of our modeling strategy, the following paragraphs will unpack these ideas and specify both the perceptual and response variables considered as well as the form of the generative models employed.

#### Perceptual variables

We considered the three following pereptual variables which refer to binary trial outcomes and are summarized in Table 2 (where win is coded as 1 and loss as 0): (i) Win/Loss Gross (WLG); this case treats real wins and fake wins as wins and near-misses as losses; (ii) Win/Loss Net (WLN); this option only considers real wins as wins and treats fake wins and near-misses as losses; (iii) Overlearn (OL), where real wins, fake wins and near-misses are all considered as wins.

**Table 2:** **Composition of perceptual and response variables for the computational modeling**.

**Composition of model variables**	**True win**	**Fake win**	**Near-miss**	**True loss**	**BI**	**DU**	**CS**	**MS**	**NS**
**PERCEPTUAL VARIABLES**									
WLN	1	0	0	0	–	–	–	–	–
OL	1	1	1	0	–	–	–	–	–
WLG	1	1	0	0	–	–	–	–	–
**RESPONSE VARIABLES**									
{BI}	–	–	–	–	1	0	0	0	0
{BI, DU}	–	–	–	–	1	1	0	0	0
{BI, DU, CS}	–	–	–	–	1	1	1	0	0
{BI, DU, CS, MS}	–	–	–	–	1	1	1	1	0

Variables are binary and composed on a trial-by-trial basis for each of combinations shown. WLG, Win/Loss Gross; WLN, Win/Loss Net; OL, Overlearn; BI, Bet increase; DU, double-up; CS, casino switch; MS, machine switch; NS, no switch (no change in behavior). Wins are encoded as 1, losses as 0.

#### Response variables

A naturalistic paradigm like ours allows for numerous readouts of behavior, and thus, many possible response variables. Here, we consider several combinations of readouts as candidate response variables. As our intention is to explain how impulsivity is manifested in a gambling paradigm, we use the BIS-11 to inform the choice of response variables. Across factor analyses of the BIS-11, conducted by Barratt (1985) and Patton et al. (1995), respectively, the 2 second-order subscales, which were found consistently include Motor Impulsiveness (the inclination to act spontaneously and aimlessly) and Non-planning Impulsiveness (the lack of future orientation and consideration of risks). Guided by these two BIS-11 subscales, we focus on four candidate response variables, bet increase (BI), double-up (DU), casino switches (CS), and machine switches (MS). Specifically, we considered nested combinations of these actions as response variables (see Table 2 and **Figure 5**). BI and DU reflect Non-planning Impulsiveness, whereas CS and MS are best characterized by Motor Impulsiveness. Quantitatively, we represent the players' trial-by-trial responses for each of these variables in a binary fashion and apply a boolean OR operator to the respective response set (Table 2).

The reinforcement-learning and Bayesian models we consider below link the expression of the above responses to the agent's internal beliefs and their uncertainy. Simply speaking, we are modeling an agent for whom stronger beliefs of winning lead to increasingly risk-seeking behavior and, at the same time, result in an increasing frequency of erratic and sensation-seeking behavior in terms of CS and MS. Critically, this probabilistic link between beliefs and actions is governed by a subject-specific parameter which, in some of the models described below, becomes a function of the agent's trial-wise uncertainty.

### Core models

#### Hierarchical Gaussian Filtering

**Figure 3:**
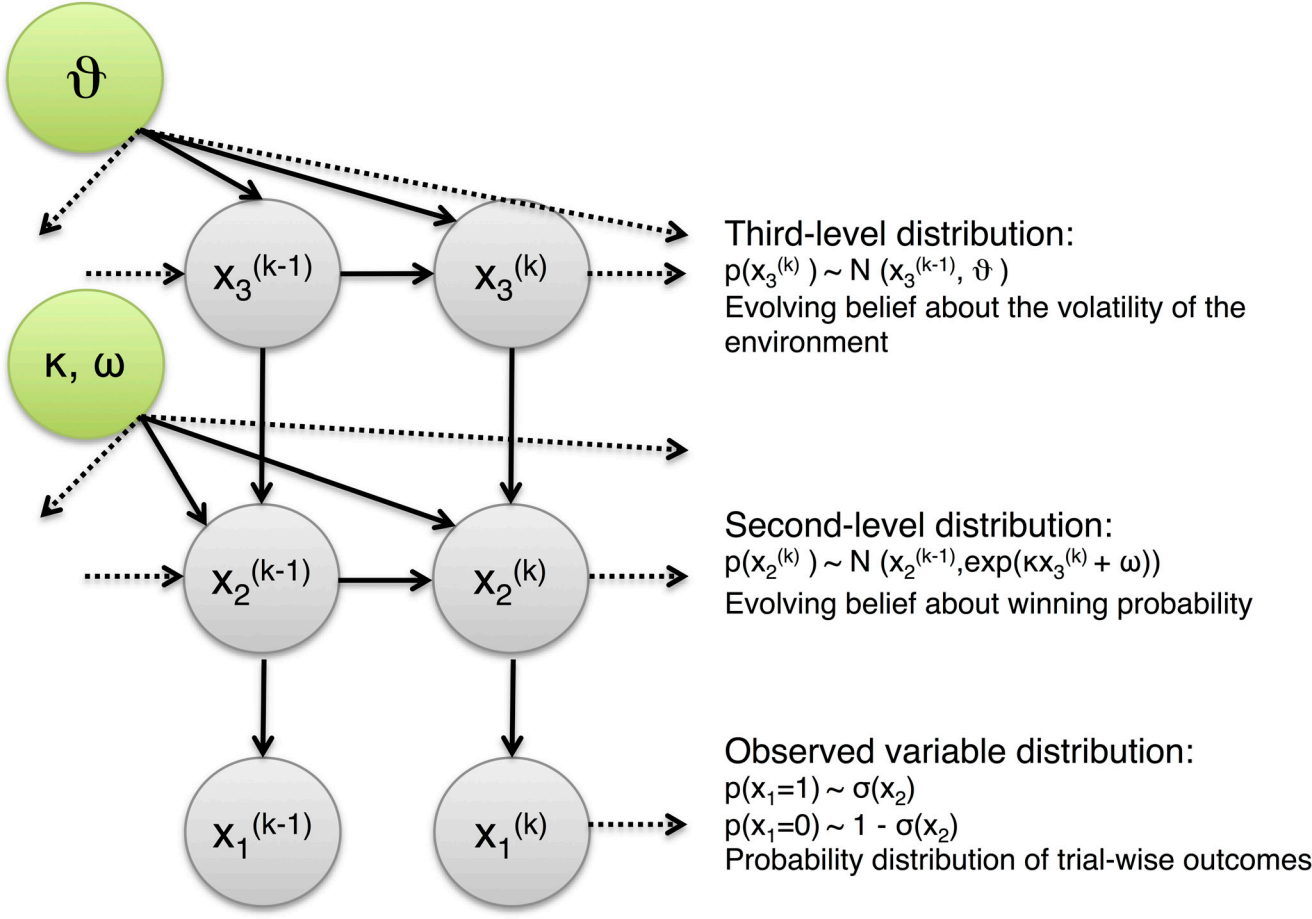
**Schematic of Hierarchical Gaussian Filter (HGF).** Different levels of the hierarchy encode a subject's estimates of different characteristics of environmental uncertainty. The first level, *x*_1_, follows the trajectory of the perceived variable in the environment, in the absence of perceptual noise. The second level, *x*_2_, tracks the probability of trial outcomes over the course of the paradigm. The step-size of the random walk by *x*_2_ depends on the highest level, *x*_3_, that tracks the global volatility of the environment. This three-level system underlies the player's belief-updating process during the game.

As motivated in the Introduction, this paper adopts a Bayesian perspective on gambling. Specifically, we use a hierarchical Bayesian belief-updating model, the HGF shown in Figure 3, to infer upon the underlying belief structure guiding individual gambling behavior and its relation to an individual's level of impulsivity (Mathys et al., 2011, unpublished work). The HGF represents a generic generative model of the sensory inputs an agent receives. It consists of hierarchically coupled Gaussian random walks, where this coupling is specified by subject-specific parameters.

To illustrate its general structure, let us assume that we track a quantity *x*_1_ in our environment which evolves as a Gaussian random walk. Let us now characterize the variance of this random walk as a function of a higher level, *x*_2_, which is itself a Gaussian random walk. *x*_2_ now controls the step size of the random walk performed at the first level via some transformation function *f*, and thus determines our uncertainty about *x*_1_. We can continue this hierarchical coupling up to some *n*-th level:

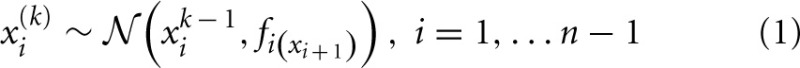



where a parameter ϑ determines the step-size on the highest level *n*:

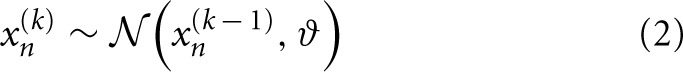



#### Perceptual model of the HGF

Figure 3 shows the graphical model and the equations of a standard three-level HGF which we are using for the present analyses. In this model, the lowest level, *x*_1_, corresponds to the perceived variable (e.g., a win), barring sensory noise (which is negligible in our case). The second level represents the evolution of the probability of trial outcomes over time. Critically, its variance depends on the third level which, in turn, represents the stability of the environment (log-volatility). In our context, this model describes how the player updates his beliefs about trial outcome probabilities under the influence of a higher belief of how these probabilities change in time (i.e., whether the slot machine is stable or volatile).

By linking beliefs to choices via a response model, we can invert this model given measured responses; for details, see Mathys et al. (2011, unpublished work). This model inversion allows for inference on subject-specific model parameters, and thus, on an individual's hierarchical belief trajectories and their associated uncertainties. Notably, the posterior estimates of subject-specific model parameters describe an individual's approximation to Bayes-optimal behavior.

In the HGF, model inversion rests on a variational approximation to full Bayesian learning which results in simple analytical belief update equations (for a detailed derivation, see Mathys et al., 2011). Intuitively, one would imagine that a belief update occurs when an agent compares the predicted to the actual sensory input, calculates an error term, and then back-propagates this error up the hierarchy to adjust beliefs at all levels. In the HGF, this occurs by passing back a prediction error that is weighted by precision (inverse uncertainty). This precision term is (proportional to) the inverse step size of the Gaussian random walk on different levels.

The belief update equations generalize to the following form: At any level *i* of the hierarchy, the belief on trial *k* (posterior mean μ^(*k*)^_*i*_ of the state *x_i_*) is updated in proportion to a precision-weighted prediction error ε^(*k*)^_*i*_; this is the product of the prediction error δ^(*k*)^_*i* − 1_ from the level below and a precision ratio Ψ^(*k*)^_*i*_:

(3) $$\mu_{i}^{\left(k\;+\;1\right)}-\mu_{i}^{\left(k\right)}\propto \Psi_{i}^{\left(k\right)}\delta_{i\;-\;1}^{\left(k\right)}=\varepsilon_{i}^{\left(k\right)}$$

where

(4) $$\Psi_{i}^{\left(k\right)}=\frac{{\hat{\pi }}_{i\;-\;1}^{\left(k\right)}}{\pi_{i}^{\left(k\right)}}$$

Here, ${\hat{\pi }}_{i\;-\;1}^{\left(k\right)}$ represents the precision of the prediction about input from the level below, and π^(*k*)^_*i*_ represents the precision of the belief at the current level. Finally, the prediction error δ^*k*^_*i* − 1_ is simply the difference between the actual value of a state (e.g., stimulus at the lowest level of the hierarchy) and our expectation of its value:

(5) $$\delta_{i}^{k}\overset{\text{def}}{=}\mu_{i}^{k}-{\hat{\mu }}_{i}^{k}$$

The equations above show that our network updates in a manner similar to RL, in which the model trains on the error signal between model predictions and observed data. A key difference, however, is that the HGF not only provides estimates of states, but also of their uncertainty (posterior variance or precision); enabling a precision-weighting of prediction errors. This precision weighting means that prediction errors lead to greater updates the more precise (less uncertain) predictions are. The HGF thus takes into account estimates of uncertainty about the hidden hierarchically related processes which generate sensory inputs. The detailed update equations for precisions (or uncertainties) can be found in Mathys et al. (2011).

#### What do the parameters mean?

The HGF described above represents a Bayesian player who updates his beliefs about trial outcome probabilities (at the 2nd level) under the influence of a higher belief (at the 3rd level) of how these probabilities change in time, i.e., whether the slot machine is currently stable or volatile. This model has four parameters of interest: three perceptual parameters (κ, ω, ϑ), and one response parameter (β) which is described below.

κ and ω determine the step size of the random walk on the second level of our hierarchy. Both of them contribute different aspects of volatility: while ω is a fixed component of step size variance at the second level, κ scales the influence of the third level on the step size variance of the second level and can thus be seen as a mediator for the dynamic component of volatility. The analyses presented in this paper fix κ to unity, because of identifiability problems that arise under some of the response models chosen here. ϑ determines the step-size of the random walk on the third level; in a sense, it represents an agent's *a priori* belief on the precision of his own inference at the second level. Collectively, these subject-specific model parameters describe the coupling of belief updates across levels and thus an individual approximation to Bayes-optimal behavior.

#### Response model of the HGF

To model the players' responses we use a softmax function, in which we vary the nature of the decision temperature, β, in Equation 6. This function describes a sigmoidal mapping from the gambler's beliefs to his chosen action:

(6) $$p(y)=\frac{1}{\left(1+\exp\text{​}\left(-2\beta \cdot {\hat{x}}_{1}\right)\right)}$$

The free parameter β encodes the curvature of the softmax, and thus decision noise, ${\hat{x}}_{1}$ is the present prediction of trial outcome probability, and *y* is the binary response variable. An intuitive interpretation of β is that it specifies how deterministically a subject's actions follow from his/her beliefs. The larger β, the steeper the softmax curve, increasingly resembling a step function, and thus the more deterministic the relation between beliefs and actions. Conversely, as β gets smaller decisions become less determined by beliefs, i.e., choices become more stochastic or exploratory.

**Figure 4:**
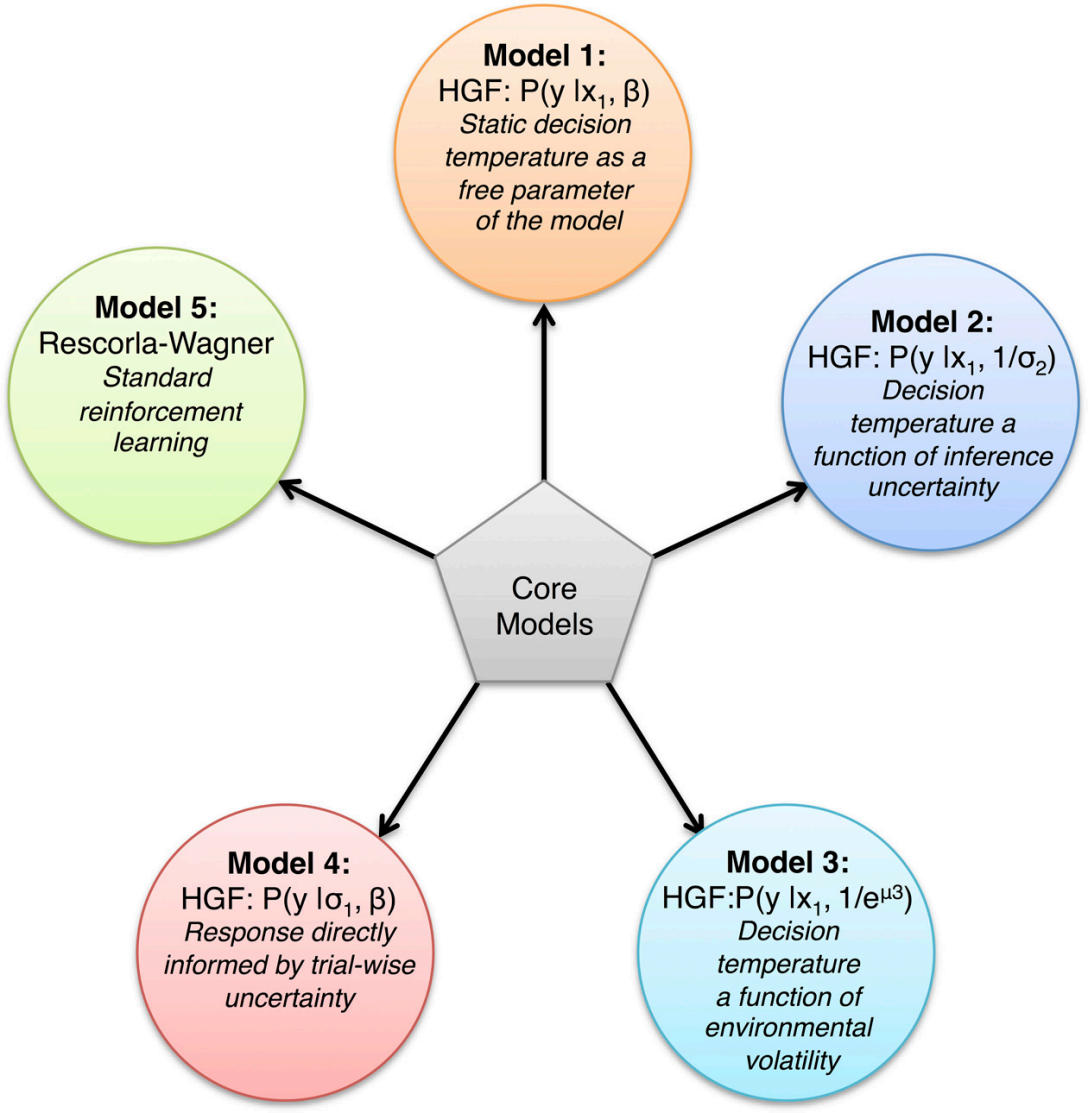
**Overview of the 5 core models tested.** Model 1–4 are different types of the HGF that differ in the response model used, whereas Model 5 is a classical Rescorla-Wagner Model with a standard softmax response function.

We consider four classes of response models that will be tested on each of the four aggregate response variables (see section Response Variables). Here, we vary the nature of β (Models 1–3) or the argument of our softmax response function (Model 4) (see Figure 4):

- Model 1: β^(*k*)^ = constant, the decision noise is a subject-specific, static feature which is independent of any higher level beliefs and which is estimated as a free parameter. The response model is then *p*(*y* = 1) = softmax(*x*_1_, β).
- Model 2: β^(*k*)^ = 1/σ^(*k*)^_2_, where σ^(*k*)^_2_ is the trial-wise uncertainty (about winning probability) on the second level of the perceptual model. The response model is then *p*(*y* = 1) = softmax(*x*_1_, 1/σ_2_).
- Model 3: β^(*k*)^ = 1/exp(μ^(*k*)^_3_), where μ_3_ is the log-volatility. The response model is then *p*(*y* = 1) = softmax(*x*_1_, 1/exp(μ_3_)).
- Model 4: *p*(*y* = 1) = softmax(4σ_1_, β), where σ_1_is the trial-wise uncertainty about winning probability on the first level of the perceptual model, and β is a free parameter as in Model 1.

Using a fixed value for β as in Model 1 is the standard formulation by which computational models employ the softmax function. By contrast, in Models 2 and 3, β turns into a dynamic quantity, which depends on trial-wise estimates of uncertainty (about the estimating winning probability, Model 2, and log-volatility, Model 3). This model space is motivated by the following rationale: both σ_2_ and *exp*(μ_3_) encode for aspects of uncertainty in the perceptual model, and higher uncertainty about one's estimates should lead to less reliance on one's beliefs when choosing a decision; i.e., more exploratory behavior. This would be expressed by a more gentle slope of the softmax curve and a lower β-value. Conversely, low uncertainty about one's estimates should map onto a steeper, more deterministic softmax curve which corresponds to a higher β-value. For this reason, σ_2_ and *exp*(μ_3_) both enter the decision model as inverses. Finally, Model 4 was inspired by a response model in Vossel et al. (2014) but formulated slightly differently. Here, we imagine an agent who is more sensation-seeking, erratic and risk-taking the higher his trial-wise uncertainty about winning probability. This uncertainty corresponds to the variance of a Bernoulli distribution at the first level of the perceptual model and takes a maximum value of 0.25 for ${\hat{x}}_{1}$ = 0.5. Using a scaling factor of 4 ensures that this argument enters the softmax appropriately, such that the maximum leads to the greatest probability of eliciting a response.

All HGF analyses were done in the context of the HGF Toolbox Version 2.1 which is freely available as part of the open source software package TAPAS (http://www.translationalneuromodeling.org/tapas/).

#### Reinforcement learning model

While hierarchical Bayesian learning is an appealing model to describe belief updating during gambling, we need to evaluate its suitability in comparison to simpler (non-hierarchical) models (Model 5, Figure 4). In particular, this includes RL models which have found application in some analyses of gambling tasks (e.g., Oya et al., 2005; Kafidindi and Bowman, 2007). Having said this, we are not aware of any RL analyses of trial-wise data from casino slot machine gambling. Here, we focus on one of the most generic and widely used RL models, the Rescorla–Wagner (RW) learning model (Rescorla and Wagner, 1972).

The RW model is a trial-wise learning model, originally developed for estimating associative learning mechanisms in conditioning. It is also frequently used in a reduced form, for example, for estimating on-line the probability of a trial-wise outcome; this is the form we use here. Updates are governed by prediction errors, scaled by a fixed learning rate:

(7) $$V^{\left(k\right)}=V^{\left(k\;-\;1\right)}+\alpha \text{​}\left(\lambda^{\left(k\right)}-V^{\left(k\;-\;1\right)}\right)$$

where *V* is the estimate of probability (of a specific outcome in trial *k*), α is known as the learning rate, and λ is actual outcome.

Here, we use the RW model as a perceptual model and combine it with a standard softmax function with a free parameter β encoding decision noise (see Model 1 above).

### Model selection

In this paper, we adopt a two-stage model selection procedure that evaluates different models with regard to two things: how well a model explains a given set of perceptual and response data features (step 1—BMS), and for which of these different data features the parameters of an optimal model best predict an external measure of impulsivity (step 2—construct validity).

#### Model selection stage 1—Bayesian model comparison

**Figure 5:**
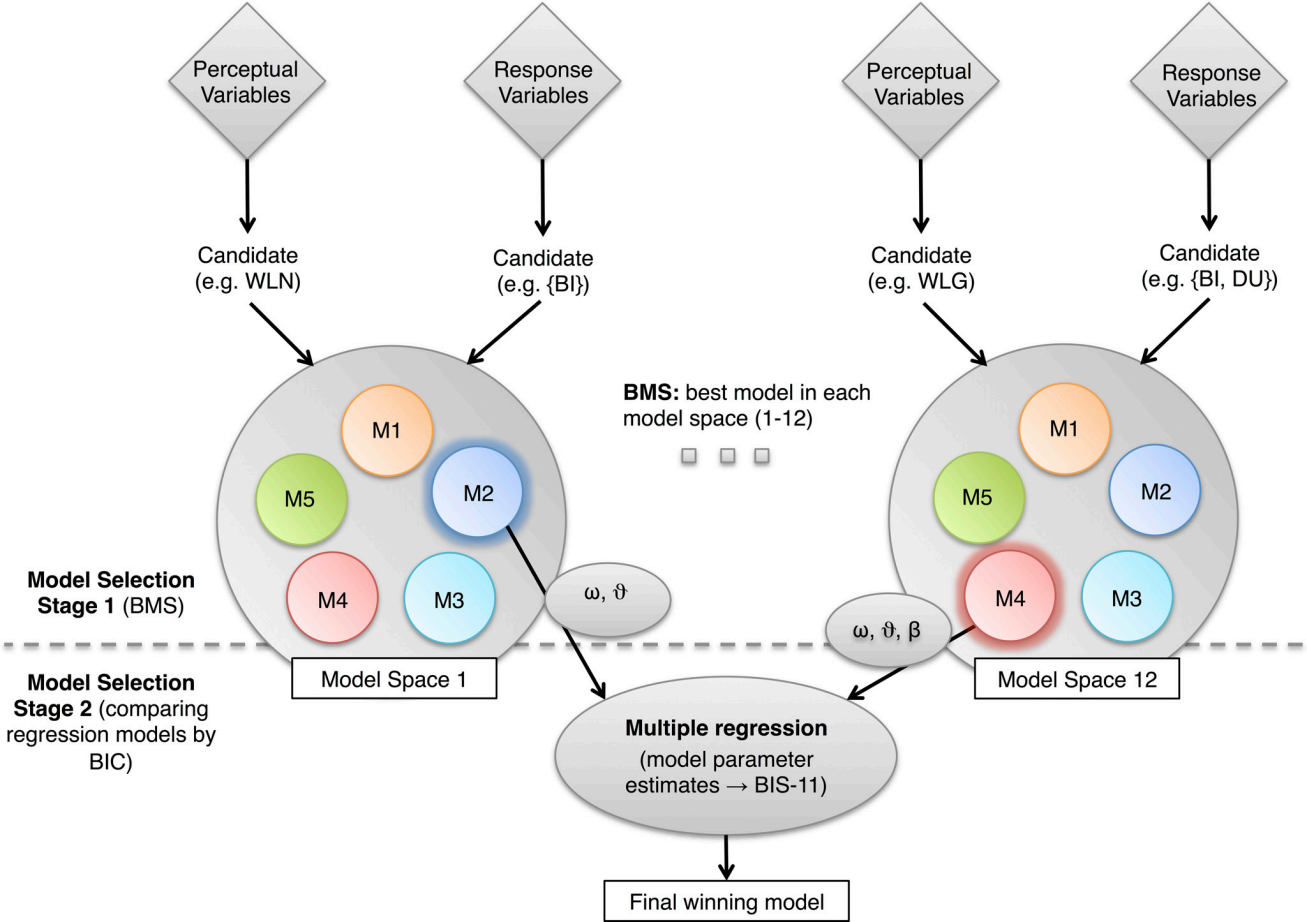
**Model selection stage 1.** For each pairing of a perceptual variable with a response variable, Bayesian model comparison was performed, yielding an optimal model (penumbra) for this combination of perceptual/response data features. This optimal model then entered stage 2 (construct validation). **Model selection stage 2**. To determine the best model with respect to an external measure of impulsivity, we regressed individual BIS-11 scores on model parameter estimates from the 12 models (one for each pair of perceptual variable and response variable) provided by model selection stage 1. The winning model is picked using a BIC comparison across regression models, to account for differing model complexities. BIS-11, Barratt Impulsiveness Scale; WLN, Net Win/Loss; BI, bet increase; DU, double-up.

As described above, first, we consider five different “core models” (Figure 4), each of which combines a particular perceptual and a particular response model. These five core models are inverted using 12 different sets of data features, which result from combining three alternative perceptual variables with four alternative response variables (**Table 4** and Figure 5). The best of the five core models for a given dataset is selected via Bayesian model comparison. This rests on the log evidence, a principled index of a model's trade-off between fit and complexity (MacKay, 2003). Critically, BMS implementations exist which can deal with heterogeneity across subjects and enable proper random effects group-level inference (Stephan et al., 2009).

The approach we employ in the present analyses is that of approximating the log evidence by negative free energy. The free energy is an upper bound approximation to the agent's surprise about seeing the data and, in contrast to the log-evidence which is analytically intractable for all but the simplest models, can be computed as part of model inversion by means of variational Bayesian (VB) optimization (see Mathys et al., 2011, for the procedure used by the HGF). For further details on model comparison using free energy, please see Penny (2012) and Stephan et al. (2009).

#### Model selection stage 2—construct validation against external criteria

Having selected an optimal model for each of the 12 sets of data features, we can evaluate the models' construct validity, i.e., how well they predict an external measure of impulsivity. For this purpose, we use the independent questionnaire scores of impulsivity (BIS-11) and perform multiple regression analyses on the model parameter estimates. In this case of competing predictions based on multiple regression models, potential differences in model complexity (due to differences in the number of generative model parameters and thus number of resulting regressors) can be corrected using the BIC. The significance of the ensuing best prediction is adjusted for multiple tests using Bonferroni correction.

## Results

### Impulsivity expressed in slot machine gambling behavior

We used a multiple regression analysis to predict impulsivity from the behavioral read-outs of the slot machine game (BI%, DU%, MS%, CS%). Together, these behavioral measures explained 32% of the variance in the individuals' BIS-11 scores [*F*_(3, 46)_ = 4.72, *p* < 0.001]. Individually, only BI percentage was significant (see Table 3); this also survived Bonferroni correction.

**Table 3:** **Multiple regression analysis for BIS-11 total scores and behavior**.

**Independent Variables**	***R*^2^**	***F*(*p*-value)**	**BIC**	***B***	***b***	***T***	***p*-value**
BI%				67.34	0.40	2.78^*^	0.008
DU%				6.12	0.19	1.42	0.163
MS%				6.52	0.05	0.34	0.736
CS%				19.33	0.20	1.32	0.194
Total	0.32	4.84 (0.0027)	−29.52				

Regression of BIS-11 scores on 4 major behavioral readouts. BI%, bet increase percentage; DU%, double-up percentage; MS%, machine switch percentage; CS%, casino switch percentage; BIC, Bayesian Information Criterion; B, unstandardized regression coefficient; b, standardized regression coefficient; Bonferroni-corrected significance level: α = *0.0125*; T, T-Test; F, F-Test;

* p < 0.0125.

### Computational modeling

#### Model selection stage 1—Bayesian model comparison

**Figure 6:**
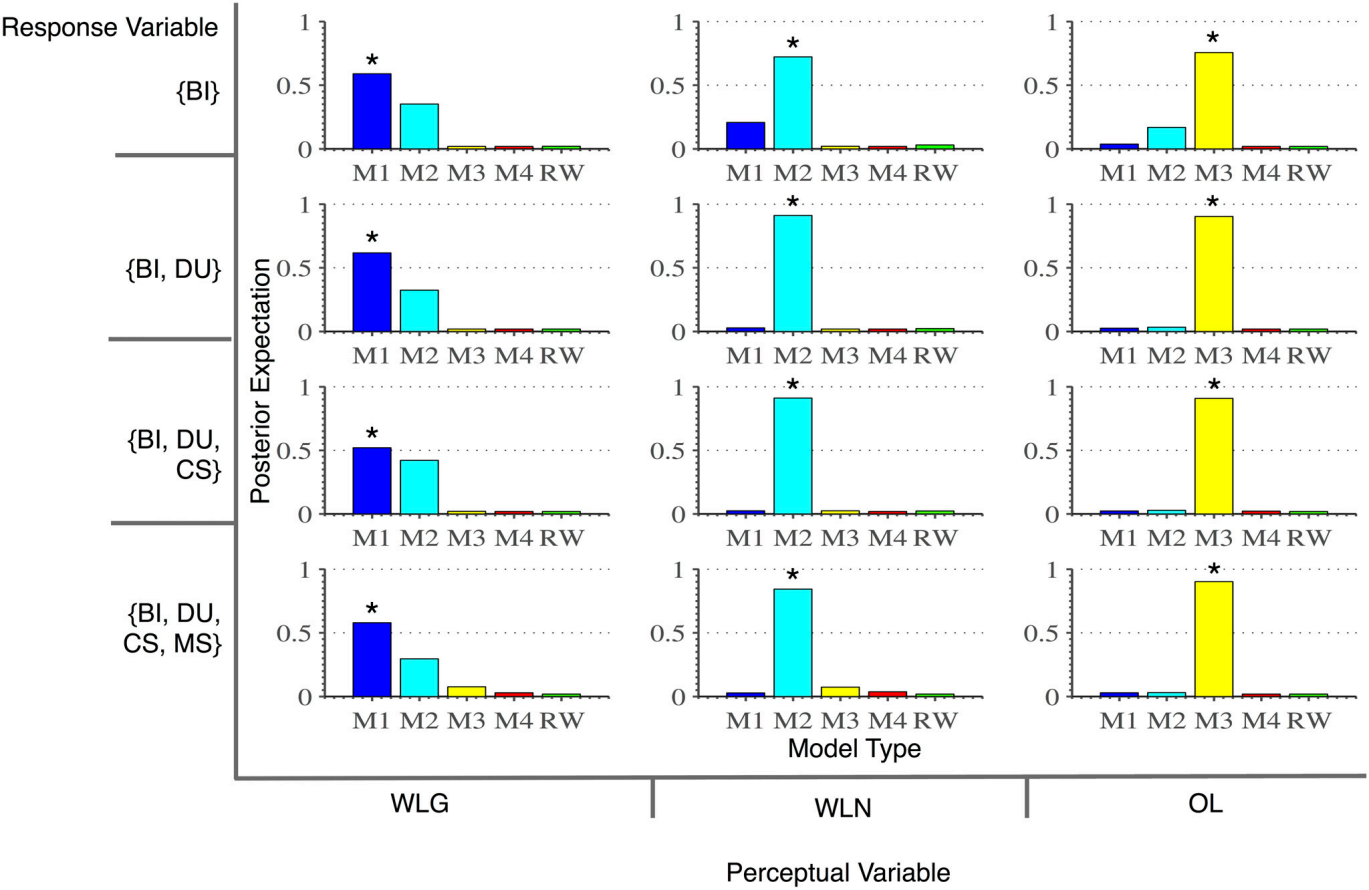
**Summary of model comparison results across all 12 classes of 5 models each (i.e., 5 optimal core models for each perceptual variable/response variable pairing).** The posterior expectation of model probability, obtained from a random effects Bayesian model selection procedure, is plotted on the y-axis. The perceptual variables span the x-axis; the response variables span the y-axis. WLG, Win/Loss Gross; WLN, Win/Loss Net; OL, Overlearn; BI, bet increase; DU, double-up; CS, casino switch; MS, machine switch; M1, M4 are the HGF core models listed in the Response Model section. RW, Rescorla–Wagner Model. The models tested in Table 4 are indicated with an asterisk. From the analysis presented, core model 2 (an HGF with second-level uncertainty instructing the decision noise in the response model) trained on perceptual variable WLN and response variable {BI, DU, CS, MS} best explains the BIS-11 scores and is the winning generative model of impulsivity.

In order to determine which of the five core models (Figure 4) best explained each of the 12 different data feature sets, resulting from combining three perceptual variables (WLG—treating real wins and fake wins as wins; WLN—treating only real wins as wins; OL—treating real wins, fake wins, and near-misses as wins; see Table 2) with four response variables ({BI}, {BI, DU}, {BI, DU, CS}, {BI, DU, CS, MS}), we used BMS. This enabled us to identify, for each of the perceptual-response feature sets, the model with the highest posterior probability (Figure 6). These selected 12 models then entered a second stage of model comparison, where we examined the construct validity of these models by testing how well their parameter estimates predicted the independent BIS-11 scores.

#### Model selection stage 2—external validation

To determine which of these 12 selected computational models best predicts impulsivity, we used multiple regression to test how well their parameter estimates predicted the BIS-11 scores (Figure 5, Table 4). As the computational models vary in the number of free parameters (e.g., some do not include a free parameter for decision temperature β) and thus the regression models differ in the number of regressors, we use the BIC to compare the regression models. We find that the core model 2 (an HGF with second-level uncertainty governing decision noise in the response model; Figure 4) with perceptual variable WLN and response variable {BI, DU, CS, MS} best explains the BIS-11 scores (highlighted in dark gray in Table 4). Two other variants of core model 2 had a similar but slightly smaller BIC value (light gray in Table 4).

**Table 4:**
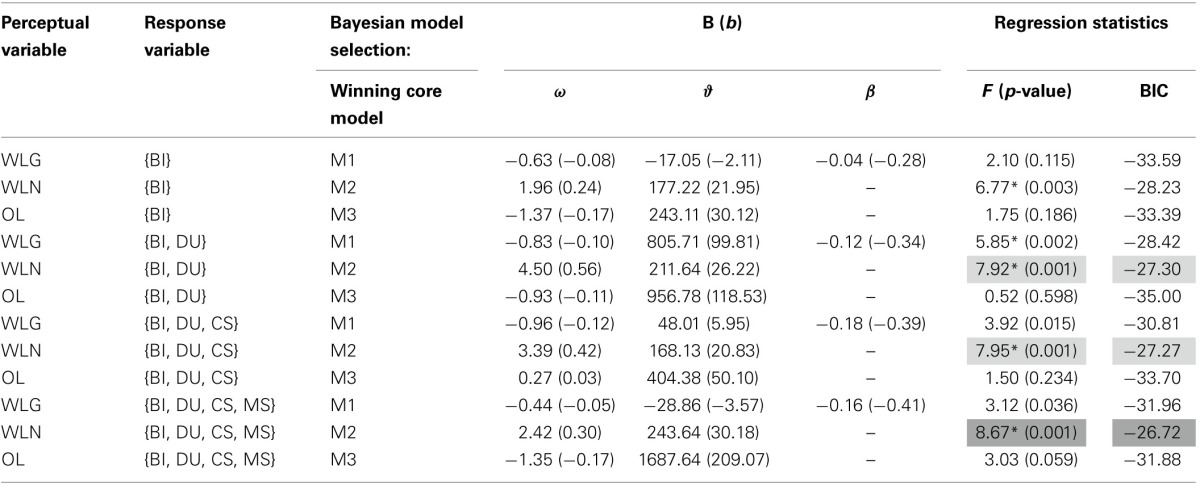
**Second-stage model comparison**.

Multiple regression of BIS-11 scores on model parameters ω and ϑ (and β, when existing). The top three models (in terms of BIC) are highlighted in gray; the best model (highest BIC) in dark gray. B, unstandardized regression coefficient; b, standardized regression coefficient; BIC, Bayesian Information Criterion; WLG, Win/Loss Gross; WLN, Win/Loss Net; OL, Overlearn; BI, Bet increase; DU, double-up; CS, casino switch; MS, machine switch; M1-3, Model 1-3; ω, the variance of the second level inference; ϑ, variance of the third level inference; β, decision temperature; ^*^Indicates significant F-test statistics (p < 0.05), Bonferroni-corrected at α = 0.004.

Together the model parameters of the winning model explained 28% of the variance in the individuals BIS-11 scores [*F*_(1, 46)_ = 8.66, *p* = 0.0007, *R*^2^ = 0.28]. Note that this model-based prediction of BIS-11 scores is significant, even after Bonferroni correction.

#### HGF parameter estimates

In a next step, we used the parameter estimates of the winning model (core model 2, perceptual variable: WLN, response Variable: {BI, DU, MS, CS}) and examined their relation to the subscales of the BIS-11, the SPSRQ, and the behavioral readouts of the game using linear regression (see Table 5, for details). Across subjects, the average posterior means of the parameters (±SD) were: ω : −4.08 ± 1.36; ϑ: 0.05 ± 0.01. Together the model parameter estimates explained 29% of the variance in Non-planning subscale scores [*F*_(1, 46)_ = 8.61, *p* < 0.006] and 15% of the variance in Motor Impulsiveness subscale scores [*F*_(1, 46)_ = 3.86, *p* < 0.029]; the latter, however, did not survive Bonferroni correction. *Post-hoc t*-tests showed that both ω [*t*_(47)_ = 2.98, *p* < 0.004] and ϑ [*t*_(47)_ = 2.10, *p* < 0.04] contribute to predicting Non-planning impulsiveness. The model parameter estimates also predict sensitivity to reward, as measured by the SPSRQ subscale [*F*_(1, 46)_ = 3.72, *p* < 0.032]; however, again this did not survive Bonferroni correction. We did not find a relation between the model parameters and Sensitivity to Punishment (SP) (Table 5).

**Table 5:** **Behavioral and BIS-11 Regressions on model parameters**.

**Dependent variables**	**B (*b*)**		***R*^2^**	***F***	***p*-value**
	**ω**	**ϑ**			
**BIS-11**					
BIS-11 Total	2.42 (0.30)	243.64 (30.18)	0.28	8.67^*^	<0.001
BIS-11: Attentional	0.36 (0.14)	57.25 (21.79)	0.08	2.03	0.143
BIS-11 Motor	0.93 (0.25)	55.65 (14.67)	0.15	3.86	0.029
BIS-11: Non-planning	1.12 (0.29)	130.75 (33.51)	0.29	8.91^*^	<0.001
**SPSRQ**					
SR	0.48 (0.12)	140.93 (36.34)	0.14	3.72	0.032
SP	−0.14 (−0.03)	95.83 (17.62)	0.02	0.44	0.645
**BEHAVIORAL READOUT**					
BI%	0.02 (0.48)	−0.54 (−0.09)	0.21	5.95	0.005
DU%	0.09 (0.47)	2.63 (0.08)	0.25	7.37^*^	0.002
MS%	0.03 (0.64)	−0.50 (−0.06)	0.40	14.44^*^	<0.001
CS%	0.04 (0.74)	0.06 (0.06)	0.57	29.42^*^	<0.001

ω and ϑ - model parameters of winning model (Model 2, Perceptual variable: WLN, Response Variable: {BI, DU, MS, CS) regressed on total BIS-11, SPSRQ and behavioral readout. B, unstandardized regression coefficient; b, standardized regression coefficient; BIS-11, Barratt Impulsiveness Scale; SPSRQ, Sensitivity to Punishment Sensitivity to Reward Questionnaire; BI%, bet increase %; DU%, double-up %; MS%, machine Switch %; CS%, casino switch %.

* Indicates significant F-test statistics (p < 0.05). Bonferroni-corrected significance level: α = 0.005.

Finally, the model parameters (ω and ϑ) predicted all main behavioral readouts from our paradigm (BI, MS, CS, and DU percentage), which survived Bonferroni correction for CS, MS, and DU (Table 5). *Post hoc t-tests* showed that there is a significant linear relationship between for CS and ω [*t*_(47)_ = 7.22, *p* < 0.001], but not ϑ [*t*_(47)_ = 0.58, *p* < 0.56].

## Discussion

This study aimed to evaluate the utility of computational modeling in characterizing slot-machine gambling behavior under realistic conditions and establish construct validity in relation to standard questionnaire measures of impulsivity. To this end, we created a naturalistic slot-machine paradigm to accrue realistic behavioral readouts from a group of healthy subjects and used a hierarchical Bayesian model of individual learning and decision-making to model the paradigm outputs.

The task builds upon previous research using slot machine tasks to explore gambling (e.g., Shao et al., 2013), but adds various degrees of freedom and realism, such as increasing the amount of the bet placed, machine switching, casino visits, and the DU option. Overall, we find that impulsivity as measured by the BIS-11 score was significantly related to an exploration of these game features. Impulsive subjects showed a stronger tendency to increase their bet size, switch between machine and casino visits and engage in a double-up option; an example of such a player is shown in Figure 2. The predictive importance of BIs for impulsivity (Table 3) is in line with studies on on-line gambling showing that gamblers with the highest levels of gambling severity exhibit the largest variance in their bet behavior (Adami et al., 2013). While the other gambling options were not correlated with BIS-11 scores when considering the “raw” behavioral data, our modeling results suggest that they jointly predict BIS-11 scores better than BIs alone (Table 4).

The mechanistic model aims at formalizing how humans solve the task at hand on a computational level. It relates potential beliefs and their evolution over time to behavioral choices. Variability across individuals within this process is captured by subject specific parameter estimates that can then be related to traits of the individual like impulsivity. To unearth this hidden information, the models have to consider (i) what information of the game players are using in order to infer their chances of winning on a trial by trial basis (perceptual variables), (ii) how they update their beliefs over time and express these beliefs through actions (core models, each representing a particular combination of perceptual and response models), and (iii) which aspect of the observed responses should be used for estimating model parameters (response variables). As described above, we use a two-step procedure that combines initial BMS (of the core model best explaining a given data set of perceptual and response variables) with subsequent construct validation through multiple regression (of parameter estimates from this selected core model on BIS-11 scores).

Concerning the first step of our procedure (BMS), Figure 6 shows that the choice of the perceptual variable has a much stronger impact on the posterior probabilities of the models than the choice of the response variable. The reason for this is simply that the perceptual variables differ much more from another than the response variables. With regard to the latter, the four response variables are nested in each other and are dominated by the frequent occurrences of BIs. By comparison, CS, and MS are less frequent and their addition to BI does not change the resulting response variable dramatically. In contrary the perceptual variable changes substantially depending on whether “fake wins” (which constitute 50% of all wins) are considered as wins or losses.

Following this procedure, the model with the highest construct validity is one which assumes that players (i) learn the Net Win/Loss probability of the game, that is, they consider only true monetary wins, (ii) update their beliefs based on their respective uncertainty on a trial by trial basis, and (iii) perform any action in the game (BI, MS, etc.) based on the belief about winning and loosing, and its respective uncertainty. It is notable that not all of our optimal core models significantly predict individual BIS-11 scores (Table 4); that is, the selection of the most informative trial-wise perceptual and response variables is crucial for predicting impulsivity by computational models.

The parameter estimates from the optimal model (ω and ϑ) significantly explain individuals' total BIS-11 score (Table 5). However, it should not be overlooked that these are in-sample predictions and the effect size estimates (i.e., *R*^2^) presented here are thus likely to be optimistic. We will address this issue in future studies with larger samples which enable out-of-sample predictions. Furthermore, when interpreting the present results, one should keep in mind that these analyses were performed in healthy volunteers who show only moderate variability with respect to BIS-11 scores (see Table 1). While this limited variance in a healthy population poses an even harder problem for statistical predictions than dealing with a highly variable population, there is no guarantee that the mechanisms highlighted by our model-based analyses will extrapolate to pathological gamblers. Instead, it is possible that qualitatively different mechanisms operate during pathological as compared to recreational gambling. This would be signaled by a different outcome of our model comparisons and will be examined in future studies with patients. Notwithstanding these caveats, the present study is important because it suggests a novel two-step modeling procedure for slot machine gambling data, and it provides concrete suggestions of which data features in slot machine gambling may be most useful for future studies.

Several of the competing models also successfully predict the BIS-11. The three leading models (highlighted in gray in Table 4), however, all share the same core model structure, in which noisy decision making is a function of perceptual uncertainty (Model 2) as well as the same perceptual input (Net Win/Loss). The models differ solely in the response variables they predict. One potential cause for that could be that some of the behavioral readouts (like CS) were relatively sparse and contributed less to the individuals' variance in gambling behavior. Finally, a variant of model 1 (which contains an additional free parameter compared to model 2) also significantly predicts BIS-11, but with a worse BIC score. Interestingly, this is the only predictive model which rests on WLG (learning from fake and true wins) as perceptual variable and a constant decision noise that is independent of the current uncertainty. Thus, this model variant might capture a general bias toward reward-related processing and behavior.

We found that subjects based their decisions on the Net Win/Loss probability, considering only real wins as wins and treating fake wins and near-misses as losses. This is not in line with an earlier study (Jensen et al., 2013) which found that subjects' estimate of winning probability increased in games with a higher number of fake wins. However, this previous study compared two slot-machines which not only differed in the number of fake wins, but also in the number of wheels (3 vs. 6), altering both game difficulty as well as the visual appearance of wins and fake wins. In our slot machine paradigm, wins and fake wins were of constant appearance; in addition subjects were informed about win magnitude after each trial, thus facilitating the distinction between real and fake wins.

Altogether, our modeling results emphasize that uncertainty plays two important roles in gambling. First, the model parameters which jointly predict BIS-11 scores (ω and ϑ) both encode aspects of uncertainty. ω represents a fixed, subject-specific tendency to change beliefs about winning probability (i.e., variance at the second level of the HGF), while ϑ determines the fluctuations of log-volatility (at the third level of the HGF) and thus the dynamic component of volatility on belief updating about winning probability. Second, the optimal response model captures a direct influence of this belief uncertainty on the individual's decision process in that decision noise is modulated by trial-wise uncertainty about winning probability. That is, the more uncertain a subject is whether he will win on the next trial the less his actions will be informed by his a priori beliefs, leading to seemingly more random behavior.

Encoding of uncertainty has previously been linked to an individual's impulsivity (Averbeck et al., 2013). While our study finds that both parameters described above jointly predict the BIS-11 score (Table 5), the different aspects of uncertainty, captured by ω and ϑ, however, have been shown to influence different parts of the brain (Iglesias et al., 2013) and may thus have differential effects on the expression of impulsivity. Indeed, our behavioral modeling analyses find a closer relation between individuals' impulsivity and the ω parameter of the model. That is, for our particular paradigm and healthy volunteers, uncertainty about winning probability appears to be more strongly related to impulsivity than the prior belief about volatility. Intriguingly this link is particularly strong for the Non-planning subscale of the BIS-11 (Table 5), suggesting that uncertainty about favorable outcomes might be a key factor in producing the lack of forethought measured by the BIS-11 (Barratt, 1985).

We used the BIS and the SPSRQ as established measures of the impulsive traits to establish construct validity of our approach (Patton et al., 1995; Torrubia et al., 2001). Having said this, the accuracy of these questionnaire-based assessments suffers from a number of limitations. PG has a high co-morbidity with mood disorders and depression, both of which tend to overshadow gambling habits and their subsequent symptoms, and may thereby cause distorted self-reports (Allcock and Grace, 1988; Black and Moyer, 1998). Further bias stems from patients lacking the requisite capacity for self-reflection (Wilson and Dunn, 2004). It has thus been suggested that interactive, computer-based neuropsychological tests provide more reliable measures of impulsivity (Kertzman et al., 2006; Chamberlain and Sahakian, 2007). Combining such tasks with a computational model of impulsivity in a naturalistic gambling setting may allow us to go even further.

Four advantages of a computational approach to such problems are particularly worth mentioning. First, computational models (i) can provide interpretations of trait like impulsivity by replacing the more descriptive nature of questionnaires with more mechanistic descriptions of how players update their beliefs during gambling and transform these into choices. In our case this is done by establishing a link between the individuals' uncertainty about winning and loosing and the resulting increase in more erratic and riskier responses. Furthermore, computational models can (ii) assess the degree of impulsiveness during actual gambling and without any need of potentially distorted self-reports, and (iii) they allow us to generate not only response traces observed in our subjects, but possible candidate response traces that reflect extreme cases of impulsive behavior. Such traces could help to identify patterns in gambling data that earmark potential problem gamblers. This approach is therefore particularly interesting for prevention with respect to online gambling. After having established a clear link between impulsivity and problem gambling, models of naturalistic play could assess the individual's impulsiveness “on the fly” and identify potential at risk players without the need of a self-report outside the actual gambling situation. Finally, (iv) the trial-wise traces of beliefs and uncertainties, inferred by a model, can serve to inform analyses of neurophysiological or fMRI data (for examples using the HGF, see Iglesias et al., 2013; Vossel et al., 2014), opening new avenues for neuroimaging research on gambling.

## Summary and outlook

The hierarchical Bayesian modeling approach presented here is capable of revealing cognitive mechanisms in gambling that are linked to traditionally defined impulsive traits of the individual. In particular, the gambling behavior of subjects, who are more impulsive, is best described by models that encode for greater uncertainty at various levels in their hierarchy, and show uncertainty-dependent coupling between beliefs about winning and subsequent decisions.

Our analyses provide a proof of concept that individual heterogeneity in gambling behavior can be quantified by computational models, enabling a mechanistic interpretation of individual gambling. Future research will have to assess the generalizability and practical utility of this approach in predicting disordered gambling behavior in various gambling settings such as online gambling.

### Conflict of interest statement

The Review Editor Dr. Harriet Brown declares that, despite being affiliated to the same institution as author Prof. Klaas E. Stephen, the review process was handled objectively and no conflict of interest exists. The authors declare that the research was conducted in the absence of any commercial or financial relationships that could be construed as a potential conflict of interest.
